# Medicine's Opportunity

**Published:** 1920-04-24

**Authors:** N. Howard Mummery

**Affiliations:** General Secretary. The Federation of Medical and Allied Societies, 5 Vere Street, Cavendish Square, London, W. 1.


### THE EDITOR'S LETTER-BOX.

**Correspondence on all subjects is invited, but we cannot in any way be responsible for the opinions expressed by our correspondents, who must give their name and address as a guarantee of good faith, but not necessarily for publication. Correspondents are reminded that brevity of style and conciseness of statement greatly facilitate early insertion.**

### MEDICINE'S OPPORTUNITY.

*To the Editor of* The Hospital.

Sir—Recent events have emphasised the urgent necessity of providing more adequate means for the collective expression of medical opinion in the councils of the nation, and, if public sympathy is not to be alienated from the cause, we must ensure that the means adopted are compatible with the dignity of our profession. The manifest unfairness of much of the public treatment meted out to us as a profession has largely, if not entirely, resulted from the failure of our various organisations to co-operate in giving expression to their views in the guidance of public opinion and in assisting to frame legislation on health matters. Whilst fully appreciating the great services many of these organisations have rendered, one has to recognise that they are only able to represent the views of their members, and that co-operation would tend to greatly increase their value to the profession and the community in general. Hitherto a few of the bodies have co-operated in times of emergency, but there has never been available a permanent conference table to which the public and the Government could turn for a united opinion on matters of national health.

This need for a central representative body, in which all branches of medical and allied practice can co-operate, has been met by the Federation of Medical and Allied Societies, which has already secured the support of some forty organisations, varying in influence from the Royal Society of Medicine to the smaller provincial medical societies, and in addition provides the co-operation of all the allied professions, as represented by the British Dental Association, the College of Nursing, Royal British Nurses' Association, Pharmaceutical Society of Great Britain, Incorporated Midwives Institute, and other bodies. Each of these organisations has representation in the Federation in accordance with its membership, and retains inviolate its individuality, freedom of action, and right to express its views separately or collectively as may seem expedient.

The Federation has already been privileged to render service in linking up existing bodies and in voicing their demand for a public inquiry into the conditions of medical service under the Insurance Acts. Its utility is much increased by the fact that seven medical Members of Parliament have seats on its Council and, as its position becomes stronger, it will be able to render increasing assistance to medical candidates, irrespective of party, and thus materially help in increasing medical representation in the House of Commons.

The Council of the Federation is now able to give effect to one of the original terms of its reference by admitting, as Individual Associates, members of the medical and dental professions. Circulars are being sent out inviting applications for such membership, at an inclusive subscription of half a guinea, and it is hoped that all medical men will contribute the small sum asked and take an active part in ensuring the permanent success of a movement which provides practical means for the collective expression of medical opinion in guiding legislation through the difficulties inseparable from the reconstruction of the health services of the country.

Yours faithfully,
N. Howard Mummery,
General Secretary.

The Federation of Medical and Allied Societies,
5 Vere Street, Cavendish Square,
London, W. 1.

